# Measurement of immune cell-derived volatile organic compounds from ex vivo and in vitro cultures: a scoping review

**DOI:** 10.1007/s11306-026-02448-y

**Published:** 2026-05-16

**Authors:** Nader Habib Bedwani, Philip Kwan Hung Leung, Jane Zen, Yuki Agarwala, Jessica Strid, George Bushra Hanna

**Affiliations:** 1 Department of Surgery and Cancer, Imperial College London, Hammersmith Hospital, Du Cane Road, Commonwealth Building, Hammersmith Campus, London, W12 0NN UK; 2 Department of Immunology and Inflammation, Imperial College London, Hammersmith Hospital, Du Cane Road, London, W12 0NN UK

**Keywords:** Volatile organic compounds, Volatilomics, Immune cells, Headspace analysis

## Abstract

**Background:**

Volatile organic compounds (VOCs) originate from cellular metabolic activity and disease-related biochemical processes and are emerging as non-invasive biomarkers. Although immune cells undergo marked metabolic and functional reprogramming during activation and differentiation, their contribution to the cellular volatile metabolome remains poorly characterised.

**Aim of review:**

This scoping review aims to systematically map experimental studies reporting untargeted VOCs emitted from mammalian immune cell cultures, with particular emphasis on volatilomics workflows and confidence in metabolite identification.

**Key scientific concepts of review:**

A systematic literature search identified experimental studies analysing headspace VOCs from primary and immortalised immune cells. Data were extracted on cell models, stimulation conditions, headspace sampling strategies, analytical platforms and data processing workflows. Analytical quality and confidence in compound assignment were assessed using the Chemical Analysis Working Group–Metabolomics Standards Initiative (CAWG-MSI) criteria. Eleven studies met the inclusion criteria, employing a heterogeneous range of sampling and analytical approaches, including solid-phase microextraction, sorbent-based thermal desorption and secondary electrospray ionisation coupled to high-resolution mass spectrometry. Across studies, reported VOC profiles were able to distinguish immune cell type, activation state and external stimuli, supporting the biological plausibility of immune-derived volatilomic signatures. However, substantial methodological variability was evident. Only one study achieved CAWG-MSI level 1 identification, and most lacked key metadata, internal standards or validation procedures. Overall, immune cells appear to emit distinct VOC signatures linked to immunometabolic state, but current practices limit reproducibility, cross-study comparability and confident biological interpretation. This review identifies metabolomics-specific methodological priorities required to characterise immune-derived VOCs.

**Supplementary Information:**

The online version contains supplementary material available at 10.1007/s11306-026-02448-y.

## Introduction

Volatile organic compounds (VOCs) are low molecular weight carbon-based chemicals that readily vaporise at room temperature (293.15 K) and can be detected in exhaled breath and other biological matrices (Drabińska et al., 2021; Einoch Amor et al., 2023; Kamal et al., 2021; Markar et al., 2018; Wen et al., 2023). These compounds are increasingly recognised as non-invasive biomarkers of physiological and pathological processes, including infection (Kamal et al., 2021), inflammation (Brekelmans et al., 2016), and malignancy (Markar et al., 2018; Woodfield et al., 2022). VOCs originate from a range of endogenous and exogenous sources including host cells, the microbiome, diet and environment (Amann et al., 2014; de Lacy Costello et al., 2014). Volatilomics is a branch of metabolomics that involves the analysis of VOCs. The total set of VOCs from a biological sample, whether derived from cells, tissues, or entire organisms, is referred to as the volatilome. Advances in analytical technologies have enabled detailed characterisation of the volatilome in both clinical and experimental settings, opening avenues for biomarker discovery and disease monitoring.

While substantial attention has been given to VOCs derived from tumour cells or the microbiome, there is growing evidence that immune cells themselves contribute significantly to the VOC landscape (Schleich et al., 2016). Considering that immune cells form an integral part of all tissues, and of the tumour microenvironment, understanding their VOC contribution is a key knowledge gap. However, investigating immune volatilomics has been largely neglected as immune cells are heterogenous, can be activated by subtle stimuli and experience rapid changes in their metabolic state which complicates consistent VOC analysis. Nonetheless, immune cells are potent sources of oxidative stress and when activated, release large quantities of reactive oxygen species (ROS) as part of the inflammatory response (Leto & Geiszt, 2006). This oxidative stress response can induce lipid peroxidation, a process that has been established as a source of VOCs with numerous VOCs arising as products of oxidative degradation of membrane phospholipids (Abbassi-Ghadi et al., 2020; Leung et al., 2025). Polyunsaturated fatty caids (PUFAs) are highly susceptible to oxidation due to the presence of multiple double bonds (Ratcliffe et al., 2020). In the presence of reactive oxygen species (ROS), lipid radicals form and propagate a chain reaction, ultimately leading to the accumulation and breakdown of lipid hydroperoxides into volatile aldehydes, alkanes, carboxylic acids and other small molecules (Antonowicz et al., 2021).

It follows that immune cells may be biologically plausible sources or contributors to VOCs and specific VOC profiles may be linked to specific immune activity. Investigating immune-derived VOCs may hold potential in diagnostic or prognostic biomarker discovery, particularly in diseases where changes in immune activity are central. This includes early-stage cancers, where cancer-associated immune responses may precede easily detectable tumours, monitoring response to immune modulating drugs such as immunosuppression in autoimmune diseases or organ transplantation, or immunotherapy in cancer.

To date, however, there has been no focussed synthesis of the literature investigating VOCs emitted by immune-derived VOC landscape is therefore essential to separate immune-specific signals from those of other biological sources and to define the role of immune-metabolism in regulating the overall tissue volatilome. This knowledge may help refine interpretation of organism-level volatilomic signatures and enhancing their translation into diagnostic tests that reflect immune activity. This scoping review maps experimental methods for untargeted characterisation of immune-derived VOCs, summarising cell types, techniques and key findings to guide further biomarker discovery.

## Methods

The PRISMA extension for scoping reviews (PRISMA-Scr) (Tricco et al., 2018) was used to aid protocol development and manuscript writing. The protocol for the scoping review was drafted using the Preferred Reporting Items for Systematic Reviews and Meta-analysis Protocols (PRISMA-P) (Shamseer et al., 2015). The final protocol was registered prospectively with the Open Science Framework on 8 May 2025 (https://osf.io/g2zmq).

This study is a scoping review aiming to address the following question based on the PICO framework: Which laboratory and analytical methods (Intervention) have been used to identify immune-derived VOCs from cell cultures (Population), and what VOCs have been reported (Outcome), with or without comparisons between immune cell types, activation states, or conditions (Comparator)?

Studies published up to 27th November 2025 were included in this review. No restrictions on language or publication date were imposed during the search. Studies were selected against defined inclusion and exclusion criteria (Table 1).

**Table 1:** Inclusion and exclusion criteria

	Include	Exclude
Population	- Cultured mammalian immune cells (including immortalised cell lines) - Freshly isolated immune cells and short-term functional assays - Co-cultures assessing VOC changes based on immune cell type or presence	- In vivo animal studies or ex vivo non-mammalian-derived immune cell cultures - Studies only exploring non-immune-derived VOCs
Intervention/exposure	- VOCs as markers of immune cell activity, differentiation, or cell type	- Studies not measuring VOCs - Studies not performing untargeted analysis - Studies only measuring non-immune VOCs (e.g. microbiome, non-immune cancer cells)
Comparator/context	- Non-comparative studies - Studies comparing immune subtypes, activation states, or disease states	- NIL
Outcome	- Identification and characterisation of immune-derived VOCs in vitro/ex vivo - Methods for headspace sampling of VOCs from immune cells	- Studies not reporting immune-derived VOCs
Study characteristics	- Experimental studies - No date restrictions	- Case reports - Reviews - Conference abstracts without accessible data - Non-English language papers - Full text not available

To identify eligible studies, a comprehensive search strategy was developed and applied across the following electronic databases: MEDLINE (including PubMed), EMBASE (Ovid), Web of Science, and Scopus. Relevant descriptors and controlled vocabulary, including Medical Subject Headings (MeSH) and Emtree terms, were used. No restrictions were placed on publication date or language and the final search was completed on 27 November 2025. Boolean operators AND and OR were applied to combine search terms. The full search strategy is provided in Supplementary Material 1. The results of the search were imported into EndNote citation and reference management tool (Version 21, Clarivate, Philadelphia, USA) and duplicates were automatically removed. Studies were then transferred from EndNote to Covidence systematic review software (Veritas Health Innovation, Melbourne, Australia) where further duplicates were automatically and manually removed.

### Study selection

Study selection wase conducted in two stages by three (NB, JZ, YA) independent reviewers. In Stage 1, titles and abstracts were screened to determine eligibility for full-text review. Studies were either included for full review or excluded, with justifications recorded according to predefined exclusion criteria. In Stage 2, full-text articles were assessed for final inclusion, again with reasons for exclusion documented. Each study was reviewed by two of the three reviewers (NB, JZ, YA). Any disagreements were resolved by consensus after discussion or, if needed, by consultation with a fourth reviewer (PL).

In addition to database searches, forward and backward citation tracking (snowballing) was conducted. This involves reviewing the reference lists of included studies (backward snowballing) and identifying studies that cite the included articles (forward snowballing), both assessed by two reviewers to identify additional relevant studies not captured through electronic searching.

### Data extraction

Data was extracted using a pre-formed data extraction template, piloted on 3 included studies and refined as appropriate. Data extracted from included studies included the quantity and type of immune cells used and their culture conditions, the methods used for sampling VOCs from the headspace of cultured immune cells, the analytical platforms employed for VOC detection, including GC-MS (Gas Chromatography-Mass Spectrometry), and the specific immune-derived VOCs identified. Additional outcomes included comparisons of VOC profiles across different immune cell types, activation states, or experimental conditions, as well as any reported challenges or limitations in detecting or attributing VOCs to immune cell function.

### Data analysis

Data extracted from eligible studies were analysed using narrative synthesis, consistent with scoping review methodology. A descriptive summary was compiled for each study, detailing immune cell type and quantity, culture conditions, comparison groups, headspace sampling methods, analytical platforms (e.g. GC-MS, SESI), quality control measures, and identified immune-derived VOCs. Studies were compared based on methodological features such as cell source (e.g. primary vs. immortalised lines), activation protocols, VOC capture techniques, analytical sensitivity, and presence of controls or replicates. The synthesis highlights common practices, methodological gaps, and limitations in the evidence base. Due to heterogeneity in study designs and outcomes, no statistical meta-analysis was conducted. Findings informed recommendations for standardisation and identified priorities for future research, particularly regarding reproducibility and clinical applicability of VOC detection from immune cell cultures.

### Quality assessment

Study quality was assessed using the Chemical Analysis Working Group Metabolomics Standard Initiative (CAWG-MSI) criteria (Sumner et al., 2007) to assess the confidence of compound identification across included studies. Categories ranged from level 1 (most confident with at least two orthogonal analytic data types), level 2 (one data type with spectral similarity to commercial library) and level 3 (least confident with one data type related to spectral or chemical properties.

## Results

### Study selection and overview of included studies

**Fig. 1:**
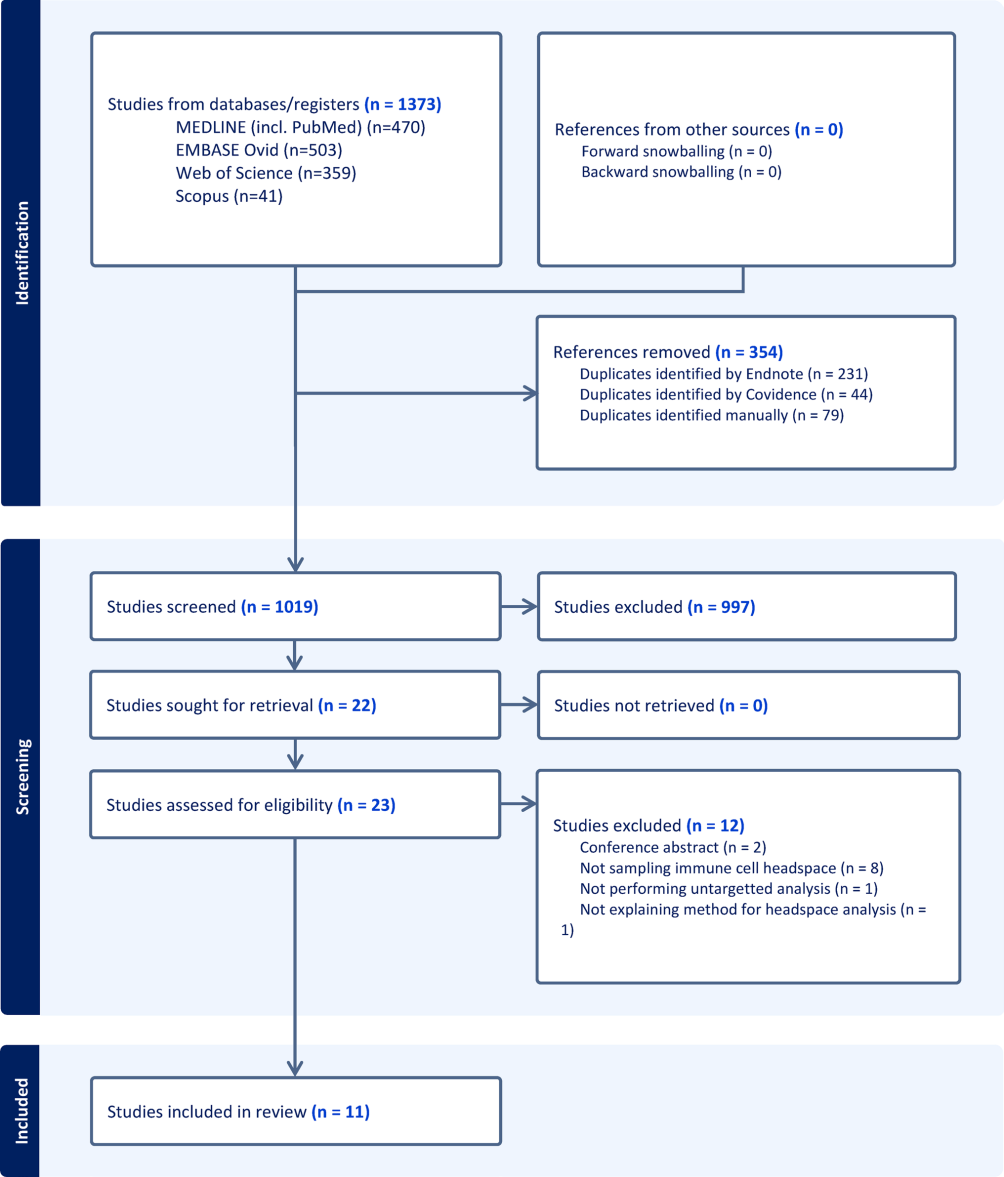
PRISMA flow diagram

A total of 11 studies (Aksenov et al., 2012, 2014; Arnold et al., 2023; Forleo et al., 2018; Hashoul et al., 2024; McCartney et al., 2019; Peltrini et al., 2024; Schleich et al., 2016; Shin et al., 2009; Tang et al., 2017; Zemánková et al., 2021) were identified for inclusion in this review. One author (Aksenov et al., 2012) was contacted for missing data but did not respond. The search and selection processes are outlined in Fig. 1. A list of excluded studies after full text review along with reason for exclusion can be reviewed in Supplementary Material 2.

### Immune cell types and culture conditions

A summary of study characteristics and immune cell cultures used can be found in Supplementary Material 3. Most studies included in this review used immortalised cell lines with some using primary murine(Arnold et al., 2023) or human (Forleo et al., 2018; McCartney et al., 2019; Schleich et al., 2016; Zemánková et al., 2021) cells or sputum samples (Peltrini et al., 2024). The objectives of the studies varied, but all reported immune cell volatilomics. Studies focussed on volatilomics of unstimulated (Aksenov et al., 2012; Hashoul et al., 2024; McCartney et al., 2019; Shin et al., 2009; Tang et al., 2017) immune cells or compared to a response to a range of stimuli including viral (Aksenov et al., 2014) and bacterial (Arnold et al., 2023; Forleo et al., 2018; Zemánková et al., 2021) infection as well as generic chemical activators (Schleich et al., 2016) or transfection of different HLA class 1 alleles (Aksenov et al., 2012). Bacterial infection was mimicked using the LPS (Forleo et al., 2018; Zemánková et al., 2021) or zymosan (Zemánková et al., 2021) in two studies while Arnold et al., (2023) used E. Coli supernatants. The number of cells in each sample was reported to be as low as 400 in one study (Aksenov et al., 2012). The range of cells used in other studies was 4 × 10^5^ (Aksenov et al., 2014) – 4 × 10^7^ (Shin et al., 2009). Three studies did not report the number of cells used (Forleo et al., 2018; Hashoul et al., 2024; Peltrini et al., 2024). Cells were cultured under standard conditions with or without the addition of antibiotics or FBS/HAS.

### Headspace sampling methods

Several different methods were used to sample cell headspace VOCs (Supplementary Material 4). Solid-phase microextraction (SPME) was commonly used (Aksenov et al., 2012, 2014; Forleo et al., 2018; Tang et al., 2017; Zemánková et al., 2021), with (Aksenov et al., 2012, 2014; Zemánková et al., 2021) or without (Forleo et al., 2018; Tang et al., 2017) the use of headspace vials. Alternative methods included direct headspace injection into SESI sampling lines (Arnold et al., 2023), collection into stainless steels canisters with cryogenic preconcentration (Shin et al., 2009) or collection onto sorbent tubes (Hashoul et al., 2024; Peltrini et al., 2024; Schleich et al., 2016) or HSSE stir bars (McCartney et al., 2019) with subsequent thermal desorption. The timing of headspace sampling after cell isolation varied between studies from immediate sampling and short-term culture over minutes, to hours or multiple sampling points over 8 days (Supplementary Material 4).

### Analytical techniques and data processing

GC-MS was the predominant analytical technique used across all studies with different configurations of columns, GC oven cycles and mass analysers such as ion trap MS, Orbitrap MS and TOF MS (Supplementary Material 4). For example, Aksenov et al., (2012) used a Varian 3800 GC with ion trap MS for compound identification, while Arnold (2023) used direct headspace sampling onto Super SESI ion source coupled with HRMS (High Resolution Mass Spectrometry) to allow real-time headspace volatilomics.

Data processing varied across studies (Supplementary Material 4) but generally included baseline correction, peak detection, retention time alignment and spectral matching to identify VOCs. Some authors using sorbent tubes excluded compounds thought to have been sourced from the sorbent material directly. Quality of compound identification varied but was typically achieved through library matching, such as the NIST (National Institute of Standards and Technology) and Wiley libraries, although differing thresholds for acceptable match quality (often ≥ 80%) may impact the accuracy and confidence of VOC identification.

### Quality assessment and control measures

Most studies included sampling from media only (Aksenov et al., 2012; Arnold et al., 2023; Forleo et al., 2018; Hashoul et al., 2024; McCartney et al., 2019; Schleich et al., 2016; Shin et al., 2009; Tang et al., 2017; Zemánková et al., 2021), empty flasks (Forleo et al., 2018; McCartney et al., 2019; Peltrini et al., 2024) or room air (Peltrini et al., 2024) (Supplementary Material 3). Where cells were activated through different means, studies used the samples as their own control (before and after activation). Most studies reported the use of biological replicates to increase reliability of their results (Supplementary Material 4). Several studies reported cell viability throughout the experiments to ensure that VOC changes could be attributed to cell activity rather than cell stress or death (Supplementary Material 4). A more comprehensive assessment using CAWG-MSI was performed (Supplementary Material 6). Among the included studies, only two reported greater than 50% of the CAWG-MSI criteria (Arnold et al., 2023; Peltrini et al., 2024) and only one achieved level 1 identification by using injected internal standards using the same TD-GC-MS method applied to samples (Peltrini et al., 2024). They compared retention times, index and mass spectra of samples with those of authenticated standards. Instrument performance was monitored daily by analysing the standard mix to calibrate retention time and index, and calibration curves were used for quantification. Of the remaining studies, one achieved level 3 metabolite identification (Tang et al., 2017) due to lack of reporting of internal standards, quality control measures or instrument calibration and limited detail on compound identification. All the remaining studies achieved level 2 by compound matching using commercially available libraries. All included studies provided relative compound quantification of which, only three performed analyses using either internal standards or normalised the results to allow for instrument variation (Arnold et al., 2023; McCartney et al., 2019; Peltrini et al., 2024).

### Headspace VOCs reported and discussion

A range of different VOCs were reported in immune cell headspace, with composition reported varying by cell type, stimulation and analytical method used (Supplementary Material 5). Several studies reported negligible VOCs from media only samples supporting their source from immune cells themselves rather than exogenous sources (Aksenov et al., 2012, 2014; Forleo et al., 2018). In unstimulated conditions, immune cells released a range of alcohols, esters, ketones and aldehydes which are consistent with lipid and amino acid metabolism. The structural diversity of baseline compounds, such as benzaldehyde isomers, alkylated phenols and aromatic hydrocarbons supports a contribution from both primary and secondary metabolic processes. Aksenov et al. (2012), genetically modified B-lymphoblastoid cells to express specific HLA class 1 alleles which resulted in unique VOCs associated with lipid peroxidation. This suggests that differential MHC expression may influence downstream metabolic pathways linking antigen presentation with the cell’s redox state.

Stimuli such as LPS, zymosan, PMA or bacterial supernatants induced VOC changes across several immune cell models. For example, Arnold et al. (2023), demonstrated that dendritic cells stimulated with E. coli-derived supernatant or LPS led to release of sulphur-containing compounds, aromatic amines and lipid-like molecules, consistent with a metabolic shift towards immune activation. The rise of 2-(methylthio)benzothiazole, not detected in unstimulated cell headspace, suggested de novo synthesis as part of the antibacterial response. Additionally, 13 C-glucose tracing experiments linked certain VOCs to glycolytic and branched-chain amino acid pathways, supporting the interpretation that VOC emission reflects changes in immune cell metabolic pathways. Similarly, infection with different influenza virus strains (Aksenov et al., 2014) led to time-dependent increase in thiirane, 1-heptanol and methyl benzoate. These compounds are associated with lipid peroxidation, oxidative stress and altered protein or cofactor use during viral replication.

In studies using primary immune cells, including eosinophils, neutrophils, monocytes and PBMCs, VOC patterns were not only stimulus-dependent but also distinct between immune cell subtypes. Schleich et al. (2016) found that PMA-activated eosinophils released higher levels of aldehydes known to be linked to lipid-peroxidation such as hexanal and heptanal, while neutrophils generated more pentane, known to be linked to oxidative-stress. In the same study, T cells were characterised by increased production of alcohols and ketones such as 2-propanol and acetone, demonstrating lineage specific volatilomic signatures. Forleo et al., (2018) reported that LPS stimulation led THP-1monoctypes to emit greater amounts of nonanal and 2-butanone, whereas PBMCs were distinguished by elevated isoprene, a by-product of cholesterol biosynthesis. This cell-type specificity contribution to headspace VOC composition supports the potential for VOCs to act as non-invasive immunophenotyping tools.

Despite the findings reported by these studies, there were several methodological limitations reducing the accuracy in comparing and interpreting the VOCs identified. Following comprehensive quality assessment for metabolomic identification, we found absolute quantification was lacking in all studies challenging comparability across experiments. Similarly, inconsistent reporting of metadata as well as variability in reported culture conditions, cell type, sampling times and instrument settings makes reproducibility and comparison across studies more difficult. The absence of standardised analytical protocols limits the generalisability of study conclusions regarding immune cell VOCs. Notably, the small number of publications exploring immune cell volatiles is in contrast of the broadening literature on exhaled breath VOCs on a range of diseases, where the immune contribution may be significant. This highlights a critical gap on mechanistic, pathway-level work linking cellular metabolism to exhaled breath VOC profiles.

#### Future directions and recommendations

To improve the quality and translatability of immune cell volatilomics, we have proposed pragmatic recommendations that align with the principles of the Metabolomics Quality Assurance and Quality Control Consortium (mQACC) (Mosley et al., 2024).

- Metadata reporting should include cell counts at the time of headspace sampling, cell viability, media composition (to include brand and lot number where applicable), headspace volume, sampling time and temperature, oxygenation conditions and the use of any antibiotics/serum as well as analytical platforms including GC column type and cycling conditions. Metadata should clearly report cell numbers seeded, incubation time post-seeding, and cell numbers at the time or after sampling to ensure accurate biomass for data interpretation.
- Specific sections detailing QC sampling strategy which should include blanks (empty headspace vials/containers), media-only/cell-free controls, spiked internal standards. Since it would be difficult to use a pooled QC for live cell samples, a minimum of 3 biological replicates should be performed, and an analytical standard mix should be analysed in a similar way to the live cells (i.e. spiked in the media and analysed periodically in the sequence) to monitor for instrumental drift.
- Daily System Suitability Testing (SST) mixtures should be performed to track retention stability, mass accuracy and sensitivity and SST metrics (e.g. retention time, drift threshold and mass accuracy) should be reported.
- If quantification assessment claims are made, then studies should include VOC identity verification by analytical standards and calibration curves (including limits of detection) and report if quantification has been absolute or relative. Where absolute quantification is not possible, semi-quantitative normalisation to internal standards and pooled QCs should be provided.
- Reporting of data processing should include parameters such as baseline correction, peak picking thresholds, deconvolution settings and library matching thresholds, including description of strategies on how library matches were verified. Raw data as well as scripts should be made available where possible.

These recommendations are intended to be achievable within analytical labs to provide a baseline to improve data quality and reproducibility. Beyond standardisation of analytical techniques, there are emerging opportunities to enhance translation of immune volatilomics. Machine learning approaches are being increasingly applied to high-dimensional VOC datasets to improve feature selection and classification as well as metabolic pathway annotation through a systems biology approach (Palma et al., 2018), although validation and standardisation to workflows is required to avoid overfitting. Isotope labelling should be used to strengthen biological interpretation linking VOCs to specific metabolic pathways. In parallel, portable sensor technologies can offer potential for point-of-care testing but would require validation of candidate VOCs prior to sensor development (Han et al., 2024). However, translation from in vitro experiments remains challenging due to matrix dilution, contributions from the microbiome or environment and compartmentalisation within the human body or metabolism of VOCs in the circulation. In vitro findings will require in vivo clinical validation in experimental models (Adam et al., 2019).

#### Complementary role of LC-MS and multi-omics

The use of paired non-volatile metabolomics (e.g. Liquid Chromatography-Mass Spectrometry (LCMS)) can support biological interpretation of VOCs to understand its mechanistic source. LC-MS may quantify metabolites both upstream or downstream of VOCs, lipid peroxidation intermediates or non-volatile carbon-based metabolites. Integrating workflows that include headspace VOC profiling with paired LC-MS and other -omics may shed insights into the biological origins of candidate VOCs through a systems biology pathway mapping approach. We encourage multi-omic pilot studies that can address this, ideally using matched samples or time-matched parallel cultures.

## Conclusion

This scoping review highlights the importance of tailoring experimental setup to the specific research hypothesis and aim. Headspace VOC profiling of immune cells has been shown to be feasible for studying immune cell activity and metabolism. The detection of VOCs in exhaled breath has been shown to be promising as a non-invasive method for monitoring health and disease. To link immune cell activity to exhaled breath VOCs, it is essential to couple in vitro immune cell volatilomics with an understanding of metabolic and biochemical pathways that lead to their release in exhaled breath. The range of experimental protocols and analytical platforms reviewed here highlights the complexity of this research area. Further studies are needed to correlate immune cell-derived VOCs with immune cell activity to understand their biological origin before correlation with those detected in breath, to enhance clinical translation.

## Supplementary Information

Below is the link to the electronic supplementary material.

Supplementary Material 1

Supplementary Material 2

Supplementary Material 3

Supplementary Material 4

Supplementary Material 5

Supplementary Material 6

## Data Availability

All data in the conduct of this review is available on request.

## Abbreviations

CAWG-MSI: Chemical analysis working group metabolomics standards initiative
eNET: Elastic net regression
FACS: Fluorescence-activated cell sorting
FBS: Foetal bovine serum
GC-MS: Gas chromatography-mass spectrometry
GC-TOF: Gas chromatography-time-of-flight mass spectrometry
HAS: Human AB serum
HLA: Human leukocyte antigen
HRMS: High resolution mass spectrometry
HSSE: Headspace sorptive extraction
LC-MS: Liquid chromatography-mass spectrometry
LLOD: Lower limits of detection
LLOQ: Lower limits of quantification
LPS: Lipopolysaccharide
MACS: Magnetic-activated cell sorting
MHC: Major histocompatibility complex
NIST: National institute of standards and technology
NR: Not reported
PBMC: Peripheral blood mononuclear cells
PBS: Phosphate buffered saline
PDMS: Polydimethylsiloxane
PMA: Phorbol 12-myristate 13-acetate
PTFE: Polytetrafluoroethylene
QC: Quality control
ROS: Reactive oxygen species
SESI: Secondary electrospray ionisation
SN: Supernatant
SPME: Solid-phase microextraction
SST: System suitability testing
TD: Thermal desorption
VOC: Volatile organic compound
WB: Whole blood
