# Supplementary material for: Measurement of immune cell-derived volatile organic compounds from ex vivo and in vitro cultures: a scoping review
Source: Metabolomics. 2026 May 16;22(3):75. doi: 10.1007/s11306-026-02448-y (PMC13179906; doi:10.1007/s11306-026-02448-y)
Supplement: Supplementary file 1 — Supplementary Material 1 [file 11306_2026_2448_MOESM1_ESM.docx]

MEDLINE (PubMed)

1. exp Volatile Organic Compounds/

2. ("volatile organic compound" OR “volatile organic compounds” OR VOC OR VOCs OR "volatile metabolite" OR “volatile metabolites” OR "gaseous metabolite" OR “gaseous metabolites” OR volatil*)

3. #1 OR #2

4. (headspace OR head-space OR "solid phase microextraction" OR SPME OR "thermal desorption" OR "gas chromatography mass spectrometry" OR GC-MS OR GCMS OR "proton transfer reaction mass spectrometry" OR PTR-MS OR "selected ion flow tube mass spectrometry" OR SIFT-MS OR "electronic nose" OR e-nose OR "gas analysis" OR "gaseous analysis" OR hisorb OR “needle trap device” OR NTD)

5. exp Immune Cells/

6. ("immune cell" OR "immune cell culture" OR “T cell” OR “T cells” OR “B cell” OR “B cells” OR leukocyt* OR lymphocyt* OR PBMC* OR “peripheral blood mononuclear” OR “NK cell” OR “NK cells” OR “natural killer cell” OR “natural killer cells” OR neutrophil* OR macrophage* OR monocyt* OR eosinophil* OR basophil*)

7. #5 OR #6

8. #3 AND #4 AND #7

EMBASE Ovid

1. ‘volatile organic compounds’/exp

2. (‘volatile organic compound’ OR ‘volatile organic compounds’ OR VOC OR VOCs OR ‘volatile metabolite’ OR ‘volatile metabolites’ OR ‘gaseous metabolite’ OR ‘gaseous metabolites’ OR volatil*).mp.

3. 1 OR 2

4. ‘gas chromatography’/exp

5. ‘solid phase microextraction’/exp

6. (headspace OR head-space OR ‘solid phase microextraction’ OR SPME OR ‘thermal desorption’ OR ‘gas chromatography mass spectrometry’ OR GC-MS OR GCMS OR ‘proton transfer reaction mass spectrometry’ OR PTR-MS OR ‘selected ion flow tube mass spectrometry’ OR SIFT-MS OR ‘electronic nose’ OR e-nose OR ‘gas analysis’ OR ‘gaseous analysis’ OR hisorb OR ‘needle trap device’ OR NTD).mp.

7. 4 OR 5 OR 6

8. ‘immune cells’/

9. (‘immune cell’ OR ‘immune cell culture’ OR ‘T cell’ OR ‘T cells’ OR ‘B cell’ OR ‘B cells’ OR leukocyt* OR lymphocyt* OR PBMC* OR ‘peripheral blood mononuclear’ OR ‘NK cell’ OR ‘NK cells’ OR ‘natural killer cell’ OR ‘natural killer cells’ OR neutrophil* OR macrophage* OR monocyt* OR eosinophil* OR basophil*).mp.

10. 8 OR 9

10. 3 AND 7 AND 10

Web of Science

1. TS=("volatile organic compound" OR “volatile organic compounds” OR VOC* OR "volatile metabolite" OR “volatile metabolites” OR "gaseous metabolite" OR “gaseous metabolites” OR volatil* OR “volatile metabolite” OR “volatile metabolites”)

2. TS=(headspace OR head-space OR "solid phase microextraction" OR SPME OR "thermal desorption" OR "gas chromatography mass spectrometry" OR GC-MS OR GCMS OR "proton transfer reaction mass spectrometry" OR PTR-MS OR “selected ion flow tube mass spectrometry" OR SIFT-MS OR electronic nose OR e-nose OR "gas analysis" OR "gaseous analysis" OR hisorb OR “needle trap device” OR NTD)

3. TS=("immune cell" OR "T cell" OR "T cells" OR "B cell" OR "B cells" OR "immune cell culture" OR leukocyt* OR lymphocyt* OR PBMC* OR "peripheral blood mononuclear” OR "NK cell" OR “NK cells” OR "natural killer cell" OR “natural killer cells” OR neutrophil* OR macrophag* OR monocyt* OR eosinophil* OR basophil)

4. 1 AND 2 AND 3

Scopus

1. TITLE-ABS-KEY(("volatile organic compound" OR “volatile organic compounds” OR VOC* OR "volatile metabolite" OR “volatile metabolites” OR "gaseous metabolite" OR “gaseous metabolites” OR volatil* OR “volatile metabolite” OR “volatile metabolites”))

2. TITLE-ABS-KEY((headspace OR head-space OR "solid phase microextraction" OR SPME OR "thermal desorption" OR "gas chromatography mass spectrometry" OR GC-MS OR GCMS OR "proton transfer reaction mass spectrometry" OR PTR-MS OR “selected ion flow tube mass spectrometry" OR SIFT-MS OR electronic nose OR e-nose OR "gas analysis" OR "gaseous analysis" OR hisorb OR “needle trap device” or NTD))

3. TITLE-ABS-KEY(("immune cell" OR "T cell*" OR "T cells" OR "B cell" OR "B cells" OR "immune cell culture" OR leukocyt* OR lymphocyt* OR PBMC* OR "peripheral blood mononuclear” OR "NK cell" OR “NK cells” OR "natural killer cell" OR “natural killer cells” OR neutrophil* OR macrophag* OR monocyt* OR eosinophil* OR basophil))

4. 1 AND 2 AND 3
