# Supplementary material for: Measurement of immune cell-derived volatile organic compounds from ex vivo and in vitro cultures: a scoping review
Source: Metabolomics. 2026 May 16;22(3):75. doi: 10.1007/s11306-026-02448-y (PMC13179906; doi:10.1007/s11306-026-02448-y)
Supplement: Supplementary file 2 — Supplementary Material 2 [file 11306_2026_2448_MOESM2_ESM.docx]

1. Aloe CA, Goh NSL, Wang H, McQualter JL, Doomun SNE, De Souza D, Pujari R, Papagianis PC, Apte SH, Barnes H, Vlahos R, McDonald CF, Hoy RF, Chambers DC, Leong TL, Bozinovski S. Iron Alters the Transcriptome and Volatile Organic Compounds in the BAL of Patients with Silicosis. Am J Respir Cell Mol Biol. 2024 Nov;71(5):617-621.

*Reason: Not explaining method for headspace analysis*

1. Ataabadi MS, Bahmanpour S, Yousefinejad S, Alaee S. Blood volatile organic compounds as potential biomarkers for poly cystic ovarian syndrome (PCOS): An animal study in the PCOS rat model. J Steroid Biochem Mol Biol. 2023 Feb;226:106215.

*Reason: Not sampling immune cell headspace*

1. Baudrexler T, Boeselt T, Li L, Bohlscheid S, Boas U, Schmid C, Rank A, Schmohl J, Koczulla R, Schmetzer HM. Volatile Phases Derived from Serum, DC, or MLC Culture Supernatants to Deduce a VOC-Based Diagnostic Profiling Strategy for Leukemic Diseases. Biomolecules. 2023 Jun 14;13(6):989.

*Reason: Not sampling immune cell headspace*

1. Infante HG, Joel SP, Warburton E, Hopley C, Hearn R, Jüliger S. Investigation of the selenium species distribution in a human B-cell lymphoma line by HPLC- and GC-ICP-MS in combination with HPLCESIMS/MS and GC-TOFMS after incubation with methylseleninic acid. Journal of Analytical Atomic Spectrometry 2007;22(8):888-896.

*Reason: Not explaining method for headspace analysis*

1. Jimenez AC, Heist CA, Navaei M, Yeago C, Roy K. Longitudinal two-dimensional gas chromatography mass spectrometry as a non-destructive at-line monitoring tool during cell manufacturing identifies volatile features correlative to cell product quality. Cytotherapy. 2022 Nov;24(11):1136-1147.

*Reason: Not sampling immune cell headspace*

1. Jüliger S, Goenaga-Infante H, Lister TA, Fitzgibbon J, Joel SP. Chemosensitization of B-cell lymphomas by methylseleninic acid involves nuclear factor-kappaB inhibition and the rapid generation of other selenium species. Cancer Res. 2007 Nov 15;67(22):10984-92.

*Reason: Not sampling immune cell headspace*

1. Lv W, Jiao X, Zhang Z, Zhang L, Song J, Wu H, Xiao J. Correlation between volatile oxidation products and inflammatory markers in docosahexanoenoic acid: Insights from OPLS-DA and predictive modelling. Food Biodsience. 2025 Jan;63():15

*Reason: Not sampling immune cell headspace*

1. McDermott C, Allshire A, van Pelt FN, Heffron JJ. Validation of a method for acute and subchronic exposure of cells in vitro to volatile organic solvents. Toxicol In Vitro. 2007 Feb;21(1):116-24.

*Reason: Not sampling immune cell headspace*

1. Neuhaus S, Seifert L, Vautz W, Nolte J, Bufe A, Peters M. Comparison of metabolites in exhaled breath and bronchoalveolar lavage fluid samples in a mouse model of asthma. J Appl Physiol (1985). 2011 Oct;111(4):1088-95.

*Reason: Not sampling immune cell headspace*

1. Schleich F, Dallinga J, Henket M, Boots A, Wouters E, Louis R, Van Schooten FJ. Eur Respir J. Conference: European Respiratory Society Annual Congress 2012;40(SUPPL.56).

*Reason: Conference abstract*

1. Schleich F, Manise M, Godschalk R, Dallinga J, Boots AW, Moonen E, Luijk K, Wouters E, Louis R, Van Schooten FJ. Am J Respir Crit Care Med. Journal of Respiratory and Critical Care Medicine. Conference: American Thoracic Society International Conference, ATS 2012;185(MeetingAbstracts)

*Reason: Conference abstract*

1. Schleich FN, Zanella D, Stefanuto PH, Bessonov K, Smolinska A, Dallinga JW, Henket M, Paulus V, Guissard F, Graff S, Moermans C, Wouters EFM, Van Steen K, van Schooten FJ, Focant JF, Louis R. Exhaled Volatile Organic Compounds Are Able to Discriminate between Neutrophilic and Eosinophilic Asthma. Am J Respir Crit Care Med. 2019 Aug 15;200(4):444-453. doi: 10.1164/rccm.201811-2210OC. PMID: 30973757.

*Reason: Not sampling immune cell headspace*
