# Supplementary material for: Measurement of immune cell-derived volatile organic compounds from ex vivo and in vitro cultures: a scoping review
Source: Metabolomics. 2026 May 16;22(3):75. doi: 10.1007/s11306-026-02448-y (PMC13179906; doi:10.1007/s11306-026-02448-y)
Supplement: Supplementary file 3 — Supplementary Material 3 [file 11306_2026_2448_MOESM3_ESM.docx]

| Study | Baseline VOCs | Differential VOCs | Biological interpretation |
| --- | --- | --- | --- |
| AkSenov 2012 | Negligible from cell media blanks (data not shown)  2-(1,1-dimethylethoxy) ethanol OR Propanoic acid, 2,2-dimethyl-, butyl ester  2-ethyl-1-hexanol  4,6-dimethyl-2-heptanone OR 2-nonanone  1-phenyl-3-buten-1-one OR Vinyl benzoate OR α-oxo-benzeneacetic acid  3-ethyl-benzaldehyde or 4-ethyl-benzaldehyde OR 2,5-dimethyl-benzaldehyde  1,3-bis(1,1-dimethylethyl)-benzene OR 1,4-bis(1,1-dimethylethyl)-benzene OR 5-tert-butyl-m-cymene  2,4-bis(1,1-dimethylethyl)-phenol OR isomers of bis(1,1-dimethylethyl)-phenol  3 unidentified compounds | Relative abundance of 10 baseline VOCs  4-bromo-2,6-di-tert-butylphenol positively identify C1RA68  Isomers of diisopropyl 1-methylene-1H-indene positively identify C1RA68 | Presence of different MHC class I compounds leads to changes in downstream metabolism |
| AkSenov 2014 | Negligible from cell media blanks and allantoic fluid  Detected in all uninfected cells:  2‑methoxy‑ethanol  Propanoic acid, ethyl ester  Butanoic acid, 2‑methyl, methyl ester  Butanoic acid, 2‑methyl, ethyl ester  Octan‑2‑one  1‑phenylbut‑1‑ene isomers  3,7‑dimethyloctan‑3‑ol  4‑ethylbenzaldehyde  Decanal  1 unidentified compound | All viral infections significantly increased abundances of these VOCs (p≤0.05). Esters and alcohols showed the largest fold‑changes  Infection-specific peaks (any duration): Thiirane, hexan‑3‑one, 5‑methyl and  heptan‑3‑one  Infection-specific peaks (24h): 1‑Heptanol and benzoic acid, methyl ester  Infection-specific peaks (48h): 3,7‑dimethyloctan‑3‑ol  Strain-specific peaks: H6N2 and H9N2 uniquely amplify 3,7‑dimethyloctan‑3‑ol and certain ketones (e.g. heptan-3-one) at 48 h | Influenza infection modifies host metabolism and signalling, driving oxidative stress, lipid peroxidation, and altered protein and cofactor use leading to strain specific changes and VOC signatures mirroring distinct stages of viral replication (e.g. lipid peroxidation leading to increased aldehydes, ketones, esters and alcohols) |
| Arnold 2023 | Example compounds: Sulphur-containing compounds (85.01060 (C4H4S), 113.00552 (C5H4OS)), oxygenated aromatics & phenolics (107.04913 (C7H6O), 95.04911 (C6H6O), 111.04404 (C6H6O2)), amines/N-containing heterocycles (136.08670 (C7H9N3), 185.10720 (C12H12N2)), fatty acid/lipid derivatives (217.14351 (C11H20O4), 225.14857 (C13H20O3)), small, oxygenated compounds/organic acids (104.03422 (C3H5O3N), 101.05971 (C5H8O2), 117.06584 (C4H8O2N2)), aromatic/aliphatic hydrocarbons (93.06986 (C7H8), 107.08549 (C8H10), 103.05419 (C8H6)), unidentified compounds (61.05580, 58.04128) | Example compounds: Sulphur-containing compounds (128.05284 (C6H9NS), 182.00908 (C8H7NS2), 114.00082 (C4H3ONS), 126.03718 (C6H7NS), 105.03686 (C4H8OS), 61.01061 (C2H4S)), oxygenated aromatics & phenolics (149.09604 (C10H12O), 121.06481 (C8H8O), 151.07529 (C9H10O2)), amines/N-containing heterocycles (179.15410 (C11H18N2), 199.12291 (C13H14N2), 171.09145 (C11H10N2), 128.02456 (C7HN3), 168.08395 (C9H13NS)), fatty acid/lipid derivatives (318.24284 (C20H31O2N), 213.18491 (C13H24O2), 189.18479 (C11H24O2), 199.13281 (C11H18O3), 195.13792 (C12H18O2)), small, oxygenated compounds/organic acids (105.03688 (C4H8OS), 112.07569 (C6H9ON), 129.09100 (C7H12O2)), aromatic/aliphatic hydrocarbons (151.14806 (C11H18), 153.16369 (C11H20)), unidentified compounds (76.03909, 81.03044, 100.03914, 126.03830)  Putative sorbitol/galacticol identified upon 24 h LPS + Glc¹³C₆ stimulation | Decrease of certain compounds supports uptake or metabolism to support cellular functions  Rise of 2‑(methylthio)benzothiazole (identified by LC-MS) in SN stimulated DCs supports de novo synthesis as part of antibacterial defence  ^13^C incorporation into lactaldehyde supports glycolytic turnover  sorbitol/galacticol isotopologues with LPS stimulation supports shifts towards known immunometabolic pathways such as branched‐chain amino acid degradation and α‑linolenic acid metabolism |
| Forleo 2017 | 2‑butanone (higher PBMC than THP-1)  styrene, cyclohexanol, cyclohexanone, 1‑hexanol‑2‑ethyl, cyclohexane and 1,1′‑(1,2‑dimethyl‑1,2‑ethanediyl)bis‑, 1,3‑di‑tert‑butylbenzene (higher in THP-1 than PBMC) | PBMC + LPS: reduced 2‑butanone, cyclohexanol, cyclohexanone and increased styrene, 1‑hexanol‑2‑ethyl, cyclohexane and 1,1′‑(1,2‑dimethyl‑1,2‑ethanediyl)bis‑, 1,3‑di‑tert‑butylbenzene  THP-1 + LPS: no change in VOCs | Elevated alcohols and ketones may reflect lipid peroxidation in cancer cells  Styrene and benzene increases may reflect oncogenic metabolism  Inflammatory stimuli (LPS) can deplete energy related compounds (2‑butanone, cyclohexanone) and increase defence associated metabolites (styrene, cyclohexane and long‑chain aliphatic species)  No change in THP-1 due to blunted cytokine responses |
| Hashoul 2023 | Headspace of media alone NR | 3-methyl-4 undecane, 2-butoxy-ethanol, 2-ethyl hexanol, hexanal, octanal, 9-methyl-1-decene, 2-butanone, butanal, oxalic acid-ethyl-2-isopropylphenyl ester, 2-phenyl-2-propanol, prenol, benzyl alcohol, benzophenone, ethyl-benzoate, 2-methyl-2-propanol, 2-ethyl-2,5-dimethyl-4-hexene-1-ol, 4-allyl-1,6,heptadiene-4-ol, oxalic acid-ethyl-2-isopropylphenyl ester, methyl vinyl ketone and mesitylene increased relative to media  Styrene, ethyl acetate, 2-ethylcyclobutanone, 4-cyanocyclohexene, benzene, dimethyl succinate, cyclopentanol, dodecane, 1-nitropropane, heneicosane, 1-butanol, propionate-2-isobutoxyethyl, acetone, propene and cyclohexanone decreased relative to media | Elevated alcohols in monocytes (especially 2‑ethyl‑1‑hexanol) relates to lipid peroxidation and membrane stability  Higher hexanal and butanal may reflect cell type specific differences in fatty acid oxidation and aldehyde metabolism |
| McCartney 2020 | Only siloxane background peaks from sorbent and low-level ubiquitous alkanes (e.g. decan, undecane) and phthalates 9e.g. diisobutyl phthylate) from media and hardware | 47 T cell‐associated VOCs with VIP > 1 of which identification was attempted for the top 20: undecane, decane, docosane, 2-ethylhexanal, 2-(2-hydroxyethoxy)ethyl acetate, 2-methyldecane, 2-ethyl-1-hexanol, benzaldehyde, 1-dodecanol, 1-methyl-4-propyl-2-pyrazoline, diisobutyl phthalate, methoxyphenyloxime plus 9 unidentified alkanes, esters, ketones and oximes | No biological interpretation offered |
| Peltrini 2024 | Headspace from blanks (no sputum) NR | Acetone, benzene, toluene, p-Xylene, a-methyl styrene, Benzothiazole (also higher in eosinophil-high, but mentioned here as a differential), benzaldehyde, decanal, nonanal, hexanal, 2-Ethylhexanal, tridecane, isothiocyanato-cyclohexane and methylene chloride reduced in eosinophil-enriched sputum  1-hexanol, styrene, phenol, decane, benzothiazole increased in eosinophil-enriched sputum | Styrene and benzothiazole linked to oxidative stress and inflammation known to occur in activated eosinophils  Aldehydes may be products of lipid peroxidation and cell membrane degradation, reflecting metabolic disturbances associated with asthma exacerbations |
| Schleich 2016 | Headspace for controls NR but identified compounds excluded from analysis | Benzylalcohol and 3-methylfuran higher in unactivated neutrophils vs neutrophils  Unidentified compound higher in unactivated eosinophils vs neutrophils  1-H-indenol and 2-butoxyethanol higher in activated neutrophils vs eosinophils  Para-dichlorobenzene higher in activated vs unactivated eosinophils  6,10-dimethyl-5,9-dodecadien-2-one higher in unactivated vs activated neutrophils | Differences in underlying cellular metabolic pathways in different cell groups and oxidative stress or lipid metabolism on activation |
| Shin 2009 | Negligible in room air  Low concentrations of acetaldehyde, hexanaldehyde styrene and 4-methyl-2-pentanone in media | Increased amounts of hexanaldehyde and acetaldehyde in cells | Likely reflects oxidative stress, lipid peroxidation and progressive cell death |
| Tang 2017 | Hexanol and cyclohexanol in blanks for SHI-1 cells; Ethanol, methylbenzene, o-xylene, dodecane, 1,3-di-tert-butylbenzene, hexanal and benzaldehyde in blanks for JEKO cells | 2,4‑dimethylheptane, benzene, 4‑methyl­decane, chloroform, 3,7‑dimethyl­dodecane and hexadecane increased in SHI-1  Hexanol and cyclohexanol decreased in SHI-1 cells  Dimethyl sulfide, 2,4-dimethylheptane, o-xylene, dodecane and 1,3-di-tert-butylbenzene increased in JEKO  Ethanol, hexanal and benzaldehyde decreased in JEKO | Altered membrane lipid metabolism and oxidative stress in malignant cells leading to alkanes, alcohols and carbonyls |
| ZemÁnkovÁ 2021 | All 91 VOCs identified in conditioned media  The following showed no change between media and cells: Propene, acetaldehyde, octene, 1,3-dioxolane, cyclohexane, benzaldehyde, limonene  3-hexanol, benzenepropanol, 1,4-dioxane, methylpropene, decanal,  tetradecane, phenylethanol, 1,2-dichlorobenzene, 2,4-dimethylphenol, cycloheptatriene, benzyl alcohol, 1,2,3-trichlorobenzene, pyridine, methylphenol, 1,4-dichloro-2-methylbenzene, 2,5-dimethylpyrazine, 1,2,3-benzotriazine, 4-Methyl-2-pentanol, 2-ethylpyridine, 3,5-dimethylphenol, 1,2-dimethylcyclohexene, 2-methylbutan-1-ol, 2,4,5-trimethylphenol, 4-ethylphenol, 3-methylthiophene, 1,2,4-trichlorobenzene, dimethylbenzene, 2-phenylethanol, ethylbenzenemethanol, benzotriazine, 2,4-dimethylphenol, 3,4-dimethylphenol, 3-buten-2-ol, 2-isopropylphenol, methylbutenol, 4,6-dimethylbenzene | Decreased in stimulated cells: Ethanol, 2-butenal, pent-2-en-1-ol, benzene, phenol, octanal, 1-penten-3-ol, 1-octen-3-one, 1,3,5-trimethylbenzene, nonanal, 2-ethyl-1-hexanol, 1,3,5-Cyclohexanetrione, toluene, hexane, 2-methylphenol, 1,3-cyclohexadiene, cyclopropanemethanol, 2-methyl-4-propylphenol, 1,4-methylphenol, 2-(2-propenyl)-4,5-dimethylphenol  Increased in stimulated cells: Acetone, furan, butan-1-ol, toluene, 1-octen-3-ol, 2-butanone, benzeneacetaldehyde, 1-pentanol, 1-butyl-2-methylbenzene, heptane, isoamyl acetate, dodecane, 1,2,4-trimethylbenzene, 2-ethylhexanol, benzophenone, 1-methyl-2-benzyl-3-butanone, benzeneethanol, 1-methyl-2-benzyl-3-butylamine, 1,2,3-trimethylbenzene, 1-hexanol, benzothiophene, 1-methyl-2-nitrobenzene, 3-methylbutan-2-one, 1,3-dimethylbenzene, benzothiophene-1-oxide | Alcohols, ketones, and hydrocarbons were predominant in the cell pellet fraction, while aldehydes, acids, and cyclic compounds were characteristic of the conditioned media fraction  Changes could reflect VOCs as signalling molecules from cell activation |

LPS = Lipopolysaccharide; MHC = Major Histocompatibility Complex; NR = Not Reported; VOC = Volatile Organic Compound
