# Supplementary material for: Measurement of immune cell-derived volatile organic compounds from ex vivo and in vitro cultures: a scoping review
Source: Metabolomics. 2026 May 16;22(3):75. doi: 10.1007/s11306-026-02448-y (PMC13179906; doi:10.1007/s11306-026-02448-y)
Supplement: Supplementary file 4 — Supplementary Material 4 [file 11306_2026_2448_MOESM4_ESM.docx]

| Study | Headspace sampling method | Sampling time point | Analytical intrumentation | Data processing technique | Quality control measures |
| --- | --- | --- | --- | --- | --- |
| AkSenov 2012 | Cells suspended in PBS and seeded into 10 mL Supelco clear glass headspace vials  Vials sealed with stainless steel screw caps and 35 (Shore A) PTFE/silicone septum  Headspace sampling using DVB/CAR/PDMS (gray hub) SPME fibres (Supelco, St. Louis, MO) with SPME fibre inserted into heated GC inlet for desorption (14 min, 240°C) | After incubation for 24 h, 37°C, 130 rpm and then transferred to 4°C to halt metabolism | Varian 3800 GC with a 4000 Ion Trap MS (Varian, Walnut Creek, CA) with an electron ionisation source (EI)  CombiPalTM robotic sample injection system (CTC Analytics, Zwingen,  Switzerland)  VF-5 ms 5% phenol/95% PDMS column (Varian)  GC column oven cycle: 1°C/min oven ramp from 5°C to 150°C, 2.5°C/min from  150°C to 200°C with 5 min holds at 50°C, 75°C, 100°C, 125°C, and 10 min holds at 150°C and 200°C; total run time 210 min  The scanned m/z range  was 35–1000 | Peak detection using moving average baseline (K=20, T=1000)  Peaks matched across samples with retention time alignment  Statistical significance assessed via Student’s t-test (p<0.05)  Compound identification through manual verification and NIST/Wiley library matching (>85% probability). | Cell viability tested using Trypan blue exclusion assay after sampling  Sampling randomised to prevent  measurement bias over time  No sequential runs  were of the same cell line |
| AkSenov 2014 | Cells seeded in uncapped 10 mL Supelco clear glass vials, sealed with rubber caps  Headspace sampling using DVB/CAR/PDMS (gray hub) SPME fibres (Supelco, St. Louis, MO) with SPME fibre inserted into heated GC inlet for desorption (5 min 50s, 240°C) | VOC sampling performed pre‑infection and post‑infection at 24 h and 48 h before transfer to 4°C to halt metabolism | Varian 3800 GC with a 4000 Ion Trap MS (Varian, Walnut Creek, CA) with an electron ionisation source (EI)  CombiPalTM robotic sample injection system (CTC Analytics, Zwingen,  Switzerland)  VF-5 ms 5% phenol/95% PDMS column (Varian)  GC column oven cycle: 1°C/min oven ramp from 5°C to 140°C, 10°C/min from  140°C to 200°C with 5 min holds at 75°C, 100°C, 125°C, 140°C and 200°C; total run time 176 min  The scanned m/z range was 0-1000 | Peak detection and alignment via MS spectral matching; statistical differences determined using Student’s t‑test (p = 0.05); compound identification via NIST/Wiley libraries (matches ≥80% accepted). | Cell viability tested using Trypan blue exclusion assay pre‑inoculation  Uninfected control vials  12 replicates per condition  Sampling randomised to prevent  measurement bias over time |
| Arnold 2023 | Four airtight culture flasks with PTFE tubing switched every 5 min via stainless‑steel valves into the SESI source  Humidified carrier gas (5% CO₂/79% N₂) flow 0.3 L/min | 1: baseline for 1 h, then for 6 h post‑SN  2: baseline for 40 min, then for 4 h post‑glucose  3: sequential 5 min per flask after 24–27 h incubation | Super SESI ion source coupled to Q‑Exactive Plus HRMS  20 μm ID nanoelectrospray capillary (Fossil IonTech, Spain) was used in studies II and III  Ionisation chamber and sampling line temperatures set to 90°C and 130°C, respectively  Capillary temperature was 275°C, sheath gas was set to 60°C, and S-lens RF level was set to 55.0  The scanned m/z range was 50-400 | Peak binning via kernel density estimation with artefacts and low-intensity peaks excluded  Statistical differences assessed using ANOVA, t-tests, and fold-change thresholds  Compound identification via molecular formula assignment using the “seven golden rules” and mnu_kegg organism library enrichment | Infusion of a certified gas‑phase standard 1 h before each run  Weekly internal/external mass calibration using lock‑mass compounds  3 biological replicates per condition |
| Forleo 2017 | Manual SPME with Supelco black‑coated fibre directly from culture flask, then desorbed in GC inlet at 250 °C | Single headspace collection after 24 h incubation | Agilent 6890N GC with ZB‑624 column with helium carrier 1 mL/min; injector 250 °C  GC column oven cycle: hold at 40°C for 1 min then ramped at 10°C/min to 65°C, then ramped at 3°C/min to 120°C, then ramped at 5°C/min to 220°C, then held for 5 min; total run time 46 min 50 seconds  The scanned m/z range was 30-500 | Enhanced Data Analysis software for chromatogram analysis and VOCs identified by matching to NIST 98 library (match >80%) with VOCC database used for data analysis | NR |
| Hashoul 2024 | Custom glass lids to petri dish with Tenax TA sorbent tubes, sealed with Teflon tape  Active sampling via disc pump at 1 mL/min for 5 min onto TD tube | Custom glass lids replaced after 4 days culture with further 1 h calibration before sampling | Thermal desorption of Tenax tubes followed by GC–MS | Chromatograms analysed using Mass Hunter qualitative analysis  Compound identification using NIST library matching (>80%)  Kruskal–Wallis & Wilcoxon tests with Bonferroni correction to identify altered VOCs | 5 biological replicates per group  Cell viability tested using Trypan blue exclusion assay before sampling |
| McCartney 2020 | Bioreactor gas exhaust line sampling collected via PTFE tubing into a 250 mL borosilicate jar containing four HSSE PDMS “Twister®” stir bars for four technical replicates | HSSE bars replaced every 24 h over 8 days | Thermal desorption at 30°C for 0.5 min then ramped at 60°C /min until 300°C then held for 3 mins into Agilent 7890A GC with DB‑5ms column  GC column oven cycle: hold at 35°C for 3 min then ramped at 30°C/min to 300°C and held for 5 min; total run time 93.8 min  The scanned m/z range was 33-300 | Deconvolution & alignment via Profinder recursive feature extraction and normalization to internal standard  Siloxane peaks removed  Autoscaled VOCs were correlated to cell density by PLS (67/33 training/validation split, venetian-blinds cross-validation) and then hierarchically clustered | Background VOCs measured with sorbent only, ‘bag and gas’ and media only controls  Twice daily measurement of viability and media conditions (nutrients, pO_2_, pH)  2 biological replicates  Dual internal standards (naphthalene‑D₈, decane‑D₂₂) and C8–C24 alkane external standard |
| Peltrini 2024 | 1 L headspace drawn from 200 mL custom-design chamber containing petri dish with sample flushed with pure air at 200mL/min  Headspace collected on TD tubes containing Carbograph 1TD and Tenax TA 60/40 | Headspace drawn from sputum within 1 hour of expectoration | Thermal desorption at 300 °C for 5 min (He 45 mL/min) onto a –10 °C cold trap, then heated to 300 °C for 5 min (He 2 mL/min, splitless) into Agilent 7820A GC  GC column oven cycle: 40 °C ramped at 5 °C/min to 300 °C and held for 5 min; total run time 57 min  The scanned m/z range was 40–450 at 3 Hz | Deconvolution and alignment using AnalyzerProPeak normalised to toluene internal standard  Siloxanes, deuterated internal standard peaks, and single-sample features removed  Elastic net regression (α = 0.5, 10-fold CV, glmnet R package) used to derive VOC signature score from 393 features across 36 samples  identification via mass spectral library matching; eNET regression with cross-validation to select canonical VOCs. | TD tubes stored at 4°C for maximum of 15 days  TD sampling with internal standard and daily calibration  Blank samples collected |
| Schleich 2016 | Closed 75 cm^2^ flasks flushed with 200 mL/min N₂ for 10 min  VOCs trapped on two-bed sorption tubes (carbograph 1TD/Carbopack X) | 0, 30, 60 and 90 minutes | Thermal desorption at 270 °C (no duration specified) onto a –5 °C cold trap, then injected onto GC column (splitless) using helium as carrier gas (flow rate not specified)  GC column oven cycle: 40 °C held for 5 min, ramped at 10 °C/min to 270 °C and held for 5 min; total run time ~33 min  The scanned m/z range was 35–350 at 5 Hz | Baseline correction, peak detection, alignment via retention indices with components in <8% samples and those with <0.15 and >2.8 retention time excluded  Compound identification using NIST library  Stepwise discriminant analysis used to build model | Cell viability measured at 90 min (95% +/- 4%) |
| Shin 2009 | Cells in Teflon vials placed in sealed glass bioreactors; flushed with low-VOC air (5% CO₂); headspace collected in evacuated stainless steel canisters | 4 h, 24 h and 48 h | Cryogenic preconcentration of 233 cm^3^ headspace into multi-column/detector GC system (He carrier gas)  GC column oven cycle and scanned m/z range NR | VOC identification via MS fragmentation matching software and compared to internal standards  Oxygenate concentrations estimated using effective carbon number scaling and linear response of flame ionisation | Blank controls collected  3 x biological replicates |
| Tang 2017 | 50 mL glass flasks with PDMS-coated SPME fiber inserted into flask headspace at 37 °C for 40 min  SPME fiber desorbed at 180 °C into GC inlet (preheated to 200 °C) | After 24 h incubation | SPME desorbed at 200°C into TRACE GC ULTRA inlet (He 1 mL/min, split mode); total run time 31 min  The scanned m/z range was 45–250 at 5 Hz | VOC peak areas normalized to CO₂ peak area  Compound identification NR | 12 x biological replicates |
| ZemÁnkovÁ 2021 | SPME headspace from 1.5 mL glass vials sealed with Teflon septa  6 different SPME coatings and sampling times tested  30 min absorption at 37 °C using optimized DVB/CAR/PDMS fiber with 50/30 μm thickness with addition of 200 mg/mL NaCl  Thermal desorption at 250°C for 5 min (He 1 mL/min, splitless), then heated to 300 °C for 5 min (He 1 mL/min, splitless) into a TG-5SilMS GC column | 2h and 24h | 1D GC–Orbitrap MS (Q Exactive GC):  GC column oven cycle: 40 °C for 5 min, ramped at 5 °C/min to 250°C, then ramped 20°C/min to 300°C and held for 5 min; total run time 45 min  The scanned m/z range was 50–500  2D GC–TOF MS (LECO Pegasus 4D):  GC column oven cycle: 40 °C for 5 min, ramped at 2 °C/min to 100°C, then ramped 15°C/min to 280°C and held for 5 min; total run time 57 min  The scanned m/z range was 29–500 at 100 Hz | Analytes identified by retention time and mass spectra using NIST2014 library  Alignment done with ChromaTOF’s Statistical Compare | Fibers conditioned daily  >90% monocyte purity by FACS  3 x biological replicates |

eNET = Elastic Net Regression; FACS = Fluorescence-Activated Cell Sorting; GC-MS = Gas Chromatography Mass-Spectrometry; GC-TOF = Gas Chromatography-Time-of-Flight Mass Spectrometry; HMRS = High Resolution Mass Spectrometry; HSSE = Headspace Sorptive Extraction; NR = Not Reported; PBS = Phosphate Buffered Saline; PDMS = Polydimethylsiloxane; PTFE = Polytetrafluoroethylene; SESI – Secondary Electrospray Ionisation; SPME = Solid-Phase Microextraction; TD = Thermal Desorption; VOC = Volatile Organic Compound
