# Supplementary material for: Measurement of immune cell-derived volatile organic compounds from ex vivo and in vitro cultures: a scoping review
Source: Metabolomics. 2026 May 16;22(3):75. doi: 10.1007/s11306-026-02448-y (PMC13179906; doi:10.1007/s11306-026-02448-y)
Supplement: Supplementary file 5 — Supplementary Material 5 [file 11306_2026_2448_MOESM5_ESM.docx]

| Study | Objective | Type of cells | Source of cells | Number of Cells | Culture conditions | Control set up |
| --- | --- | --- | --- | --- | --- | --- |
| AkSenov 2012 | Headspace VOC of cells with different HLA class 1 alleles | B-lymphoblastoid cells | Immortalised human cell line (C1R) | 400 cells in 500 μL seeded*  12 biological replicates | 37°C, 5% CO_2_, in RPMI 1640 medium  (Invitrogen, Carlsbad, CA) supplemented with HyClone 10% defined fetal bovine serum (Thermo, Waltham, MA) and gentamycin  sulfate (0.05 mg/mL; JRS, Patterson, NY).  Geneticin 0.5 mg/mL added to transfected cells  Cells suspended in PBS for headspace analysis  <3 passages | HLA class 1 negative +/- single allele transfection (A2, A69, B7 and B27)  Cell media blanks |
| Aksenov 2014 | Headspace VOC associated with influenza infection | B-lymphoblastoid cells | Immortalised human cell line (C1R) | 400,000 cells in 500 μL  12 biological replicates | Incubated at 37°C, 5% CO₂  RPMI 1640 (serum‑free with TPCK trypsin) for infection  RPMI 1640 (10% FBS and 50 µg/mL gentamicin) for VOC sampling)  Post‑infection incubation for 24 or 48 h (130 rpm shaking for VOC experiments). | Cells before and after infection with avian (MOI 10) H9N2 and H6N1, and human H1N1 (MOI 1 and 10) |
| Arnold 2023 | 1: Real‑time VOC profiling of DCs upon E. coli SN stimulation  2: ¹³C‑glucose incorporation into VOCs over 4 h  3: ¹³C‑glucose tracing ± LPS over 24 h | Dendritic cells | Murine bone‑marrow‑derived dendritic cells (BMDCs) differentiated from C57BL/6NCrl mice | 5 × 10⁶ cells in 5 mL per flask (1 × 10^7^ for DC3 in study 2) (1 mL of 66 mM glucose tracer added in study 2) | RPMI 1640 + 10% FCS (dialysed FBS for ¹³C studies), 10 ng/mL GM‑CSF, (37 °C, 5% CO₂)  LPS in study III; 2 h rest pre‑measurement | 1: SN1 vs. SN2 vs. medium control with SN stimulation 1:4 dilution  2: Glc¹²C₆ vs. Glc¹³C₆  3: Glc¹²C₆ vs. Glc¹³C₆ ± LPS |
| Forleo 2017 | Compare headspace VOC profiles of LPS‑stimulated THP‑1 vs. PBMC and vs. cell‑free medium after 24 h. | Monocytes cell line and healthy PBMCs | THP‑1 human leukaemic monocyte cell line and primary healthy human PBMCs isolated by gradient centrifugation | NR  No mention of replicates | RPMI 1640 + 2 mM glutamine, 100 µg/mL streptomycin, 100 IU/mL penicillin, 10% FBS (5% CO₂, 37 °C) | LPS (1 µg/mL) treated vs. untreated THP‑1 and PBMCs  Cell medium blanks |
| Hashoul 2024 | Profile headspace VOCs in immune cell culture  Separate objective to measure headspace VOC from transwell co-cultures or one way headspace crosstalk between immune and cancer or normal epithelial cell lines crosstalk as well as effect of coculture and one way headspace crosstalk on transcriptomics and proteomics | Pro‑monocytic myeloid cells | Immortalised human cell line (U937) | Optimised seeding density to reach 60–90% confluency in petri dish (sample volume or cell number NR) | RPMI 1640 + 10% FBS + 1% penicillin/streptomycin (37 °C, 5% CO₂)  3-5 passages | Monoculture vs media only control |
| McCartney 2020 | Monitor VOC emissions from expanding primary T cells in bioreactors  Separate objectives comparing expanding ovarian cancer cell line to T cells and comparing liquid-phase to gas phase VOCs | T cells | Primary human T cells | 7×10⁵ cells/mL in 1 L culture medium | Xuri Expansion Medium + 1% penicillin/streptomycin + 5% human AB serum + 350 IU/ml IL-2 (37 °C, 5% CO₂) | Bag and gas and media only controls  4 headspace samples per cell culture sample |
| Peltrini 2024 | Profile VOCs in sputum headspace to discover biomarkers distinguishing eosinophil-enriched vs non-eosinophil-enriched asthma sputum.  Separate objective to validate model with patient breath | Presumed eosinophils in spontaneous sputum from severe asthma patients (n = 36) collected ≥6 weeks post-exacerbation on stable treatment | Human sputum  22 eosinophil-enriched, 14 non-eosinophil-enriched (based on ≥3% sputum eosinophils) | NR | No culture; sampled directly from sputum | Background room air |
| Schleich 2016 | Discriminate VOCs from eosinophils vs. neutrophils +/- PMA for activation | Eosinophils and T cells | Primary human isolated from WB using gradient centrifugation and CD16 MACS selection from lymphocytes | 3×10^6^ cells; sample volume NR | RPMI culture medium + 2% FCS (37 °C, 5% CO2) +/- 100 ng/mL PMA | Media +/- PMA and empty flasks |
| Shin 2009 | Measure VOCs emitted by HL60 cells over time and differentiate them from background and media-only controls | Neutrophil promyelocyte | HL60 cell line | 40 x 10^6^ in 30 mL | RPMI 1640 + 10% FBS (37 °C, 5% CO₂); serum-free AIM-V media for 48 h prior to sampling | Media only and room air |
| Tang 2017 | Profile headspace VOC of lymphoma and leukaemia cells | Non-Hodgkin lymphoma, acute mononuclear cells, lymphocytes and macrophages | JEKO and SHI-1 cancer cell lines  Lymphocyte and macrophage cell lines (source NR) | 5 mL of 0.7 x 10^5^ cells/mL | RPMI-1640, 10% FBS, 100 mg/L streptomycin, 100,000 units/L penicillin, 293 mg/L L-glutamine; (37 °C, 5% CO₂); media changed every 2 days for three weeks and cells in logarithmic growth phase taken for headspace analysis | Media only |
| ZemÁnkovÁ 2021 | Profile VOCs from primary monocytes ± zymosan or LPS stimulation | Peripheral blood monocytes | Primary human isolated from WB using MACS | 2 x 10^6^ cells/mL; sample volume NR | X‑VIVO 15 medium, (37 °C, 5% CO₂) ± 5 µg/mL zymosan or 1 µg/mL LPS | Conditioned media/supernatant only |

HLA = Human Leukocyte Antigen; LPS = Lipopolysaccharide; MACS = Magnetic-Activated Cell Sorting; NR = Not Reported; PMA = Phorbol 12-myristate 13-acetate; SN = Supernatant; VOC = Volatile Organic Compound; WB = Whole Blood

*Denotes number of cells seeded prior to incubation which is not directly comparable with final samples cell numbers reported in other studies.
