# Supplementary material for: Measurement of immune cell-derived volatile organic compounds from ex vivo and in vitro cultures: a scoping review
Source: Metabolomics. 2026 May 16;22(3):75. doi: 10.1007/s11306-026-02448-y (PMC13179906; doi:10.1007/s11306-026-02448-y)
Supplement: Supplementary file 6 — Supplementary Material 6 [file 11306_2026_2448_MOESM6_ESM.docx]

| tudy | Sample preparation | | | | | Experimental analysis | | Instrumental performance | | | | Method validation | | | | | Metabolite identification | | | | | | | | | | Data processing | | Metabolite identification | |
| --- | --- | --- | --- | --- | --- | --- | --- | --- | --- | --- | --- | --- | --- | --- | --- | --- | --- | --- | --- | --- | --- | --- | --- | --- | --- | --- | --- | --- | --- | --- |
|  | Biological replicates | Cell/tissue harvesting | Cell/tissue processing | Storage conditions | Extraction method | Instrument description^*^ | Separation column | Instrument description^†^ | Sample introduction and delivery | Ionisation source | Mass analyser description and acquisition mode | m/z calibration standard^‡^ | QC samples | Internal standards used | Retention time markers | Accuracy and precision | Relative quantification | | | | | Absolute quantification | | | | | Post acquisition data pre-processing | |  |  |
|  |  |  |  |  |  |  |  |  |  |  |  |  |  |  |  |  | Description and quantifier of IS | Description of method used for assessing instrument response | Replicate analysis | Calibration curve for each metabolite | Range of standards used | Quantification of method accuracy | Quantification of method precision | LLOQ | LLOD | Recovery/stability | Data file conversion method | Data preprocessing | CAWG-MSI score | Level |
| Aksenov 2012 | Y | N/A | N/A | N | Y | Y | Y | Y | Y | Y | Y | N | N | N | N | N | N | N | Y | N | N | N | N | N | N | N | N | Y | 10 | 2 |
| Aksenov 2014 | Y | N/A | N/A | N | Y | Y | Y | Y | Y | Y | Y | N | N | N | N | N | N | N | Y | N | N | N | N | N | N | N | N | Y | 10 | 2 |
| Arnold  2023 | Y | Y | Y | N | Y | Y | Y | Y | Y | Y | Y | Y | Y | N | N | N | N | Y | Y | N | N | N | N | N | N | N | N | Y | 15 | 2 |
| Forleo  2017 | N | Y | Y | N | Y | Y | Y | Y | Y | Y | Y | N | N | N | N | N | N | N | N | N | N | N | N | N | N | N | N | Y | 9 | 2 |
| Hashoul 2023 | Y | N | N | N | Y | Y | N | Y | Y | Y | Y | N | N | N | N | N | N | N | Y | N | N | N | N | N | N | N | N | Y | 8 | 2 |
| McCartney 2020 | Y | Y | Y | N | Y | Y | Y | Y | Y | Y | Y | N | N | Y | N | N | N | Y | Y | N | N | N | N | N | N | N | N | Y | 14 | 2 |
| Peltrini 2024 | N | Y | Y | Y | Y | Y | Y | Y | Y | Y | Y | N | Y | Y | N | N | Y | Y | N | Y | Y | N | N | N | N | N | N | Y | 17 | 1 |
| Schleich 2016 | Y | Y | Y | N | Y | Y | Y | Y | Y | Y | Y | N | N | N | N | N | N | N | Y | N | N | N | N | N | N | N | N | Y | 12 | 2 |
| Shin  2009 | Y | N/A | N/A | N | Y | Y | Y | Y | Y | Y | Y | N | N | Y | N | N | N | N | Y | N | N | N | N | N | N | N | N | Y | 11 | 2 |
| Tang  2017 | Y | N/A | N/A | N | Y | Y | N | Y | Y | Y | Y | N | N | N | N | N | N | N | Y | N | N | N | N | N | N | N | N | N | 8 | 3 |
| Zemánková 2021 | Y | Y | Y | N | Y | Y | Y | Y | Y | Y | Y | N | N | N | N | N | N | N | Y | N | N | N | N | N | N | N | N | Y | 12 | 2 |

CAWG-MSI = Chemical Analysis Working Group Metabolomics Standards Initiative; LLOD = Lower Limits of Detection; LLOQ = Lower Limits of Quantification; N = No; N/A = Not Applicable; QC = Quality Control Y = Yes

^*^e.g. GC-MS or SESI-HRMS platform

^†^e.g. Column oven parameters/GC inlet conditions

^‡^Lock-mass compounds, certified gas infusion
